# A loop-mediated isothermal amplification assay for the detection and quantification of JC polyomavirus in cerebrospinal fluid: a diagnostic and clinical management tool and technique for progressive multifocal leukoencephalopathy

**DOI:** 10.1186/s12985-018-1046-z

**Published:** 2018-08-31

**Authors:** Hitomi Kinoshita, Kazuo Nakamichi, Chang-Kweng Lim, Mutsuyo Takayama-Ito, Lixin Wang, Itoe Iizuka, Ichiro Kurane, Masayuki Saijo

**Affiliations:** 1 Department of Virology 1, National Institute of Infectious Diseases, Shinjuku-ku, Tokyo, 162-8640 Japan; 2 Present Address: School of Tropical and Laboratory, Hainan Medical University, Hainan, 571199 China

**Keywords:** LAMP, PML, JC polyomavirus, Detection, Quantification

## Abstract

**Background:**

JC polyomavirus (JCV) is the causative agent of progressive multifocal leukoencephalopathy (PML), a demyelinating disease of the central nervous system in immunosuppressed patients. PML usually has a poor prognosis. Detection and quantification of the JCV genome in cerebrospinal fluid (CSF) is an efficacious tool for the diagnosis and management of PML, for which proper therapeutic interventions are required.

**Methods:**

A loop-mediated isothermal amplification (LAMP) assay was applied for the quantitative detection of JCV. The LAMP assay was evaluated for the efficacy in diagnosis of PML in comparison with the TaqMan-based quantitative real-time PCR (qPCR) assay using 153 CSF specimens collected from patients with suspected PML.

**Results:**

The LAMP assay showed no cross-reactivity against other polyomavirus plasmids, viral DNA, and viral RNA, which causes encephalitis, and detected 1 copy of the standard DNA per reaction. Among 50 qPCR-positives, 42 specimens (containing JCV genome ranged from 3.2 × 10^0^ to 3.2 × 10^6^ copies/reaction) showed positive reactions and 8 specimens (containing 0.9 to 19.9 copies/reaction) showed negative in the LAMP assay. Furthermore, 3 of 103 qPCR-negative specimens showed positive reactions in the LAMP assay. The sensitivity, specificity, positive predictive value, and negative predictive values of the LAMP assay were 84% (42/50), 97% (100/103), 93% (42/45), and 93% (100/108), respectively. The kappa statistic was 0.83. The JCV loads determined by the LAMP assay showed a strong positive correlation with those determined by the qPCR assay for 33 specimens with copy numbers of ≥1 copies/reaction (*r* = 0.89). Additionally, the LAMP assay could monitor the JCV genome copy number in CSF for sequential samples equivalently to qPCR assay.

**Conclusions:**

The newly developed LAMP assay is highly specific against JCV and detect the JCV genome in the sample DNA containing 20 or more copies of JCV genome per reaction with 100% sensitivity (*n* = 29), which corresponds to ≥3 × 10^3^ copies/mL of CSF. The LAMP assay is useful for the diagnosis and offers valuable information for the evaluation and management of PML in the clinical setting.

## Background

JC polyomavirus (JCV), a non-enveloped DNA virus, belonging to the *Polyomaviridae* family, is the causative agent of progressive multifocal leukoencephalopathy (PML), a fatal demyelinating disease of the central nervous system [1]. The JCV genome is composed of double-stranded, circular, supercoiled DNA that is 5.1 kb in length and encodes six major proteins, including three structural capsid proteins (VP1, VP2, and VP3), the nonstructural agnoprotein, and two regulatory proteins (large T and small t antigens) [1].

JCV is distributed worldwide and causes ubiquitous infections in humans. Seroprevalence studies on JCV indicate that humans are infected with JCV during childhood, and up to approximately 35–70% of the adult population is positive for the JCV antibody [1–3]. After primary infection, JCV establishes latent infection in the kidneys, bone marrow, and lymph nodes [2–4]. It is assumed that the development of PML is a result of the reactivation of JCV from latency in immunosuppressed patients, including hematopoietic stem cell transplant recipients, those with human immunodeficiency virus (HIV) infection, those with hematologic malignancies, and those treated with immunosuppressive therapy [2, 4]. JCV enters the brain and causes lytic infection of the oligodendrocytes, which results in demyelination in such patients [2]. Prompt therapeutic intervention is required, because PML progresses rapidly and the 3 month mortality rate for all cases is 30–50% [2, 5]. Individuals infected with HIV accounted for 85% of all PML patients [6, 7]. Furthermore, PML was found in approximately 4% of all HIV-infected patients before the introduction of highly active antiretroviral therapy (HAART) [7].

The detection of JCV DNA in cerebrospinal fluid (CSF) is necessary for probable PML, if a patient has suspected clinical features or neurological image findings, and definite PML requires all of clinical, imaging finding and laboratory confirmation and thus the detection of JCV genome in CSF is of great value for making diagnosis [2, 8]. Nested PCR and real-time quantitative PCR assays were developed as highly efficacious methods for the detection of JCV genome [9–20]. Several studies suggested a significant correlation between the JCV load in CSF and clinical outcomes, such as survival time, indicating that quantification of the JCV genome in CSF is of great benefit to predict prognosis [17, 20–24]. For instance, a high JCV load (> 4.8 × 10^4^ genome copies/mL CSF) is associated with poor prognosis [25].

Loop-mediated isothermal amplification (LAMP) is a unique nucleic acid amplification method that amplifies the target sequence under isothermal conditions (60–65 °C) [26]. The technique relies on the strand displacement activity of DNA polymerase and a set of four specially designed primers [26]. A set of four primers recognizes six distinct nucleotide sequences on the target genome and an additional primer, named the loop-primer (LF), which improves the efficacy of genome amplification [27]. The LAMP technique has been confirmed for the efficient, specific, and rapid amplification of target genes [26]. In addition to the detection of the target gene, the LAMP technique enables quantification of the target genome by real-time monitoring of turbidity, which is associated with the level of by-product accumulation during the amplification reaction [28]. Therefore, the LAMP assay has been applied as a simple method for the detection of several pathogens associated with viral diseases [29–32].

Here, we developed a LAMP assay for the detection and quantification of the JCV genome in CSF collected from patients with suspected PML.

## Methods

### Plasmids and viruses

A plasmid containing the whole genome sequence of JCV Mad-1 strain (pJCV1–4- > pJCV: pJCV) [33] was obtained from the Health Science Research Resources Bank (Osaka, Japan). Plasmids containing the complete genome of other polyomaviruses, including the BK virus (BKV) (pBKV34–2), simian virus 40 (SV40) (pBRSV), and murine polyomavirus (MPyV) A2 strain (pPy-1), were purchased from the American Type Culture Collection (Manassas, VA, USA). Plasmids containing the genome of human immunodeficiency virus (HIV) type 1 subtype B (pNL-432) and subtype C (pINDIE-C1) were kindly provided by Dr. Masashi Tatsumi (National Institute of Infectious Diseases [NIID], Tokyo, Japan). The following viruses were used in this study: herpes simplex virus type 1 (HSV-1) TAS strain, varicella-zoster virus (VZV) V-Oka strain (kindly provided by Dr. Naoki Inoue, NIID), Japanese encephalitis virus (JEV) JaTH160 (GenBank accession no. AB269326), West Nile virus (WNV) NY99–6922 (AB185915), lymphocytic choriomeningitis virus (LCMV) WE strain, measles virus (MeV) Schwarz FF-8 strain (kindly provided by Dr. Katsuhiro Komase, NIID), and rabies virus (RV) HEP-Flury strain. Viral DNA/RNA was extracted from 200 μL of infected culture fluid using the High Pure Viral Nucleic Acid Kit (Roche Molecular Systems, Inc., Pleasanton, CA, USA) and the QIAamp Viral RNA Mini Kit (Qiagen, Hilden, Germany) eluted with 50 μL of elution buffer.

### Primer design

**Fig. 1:**
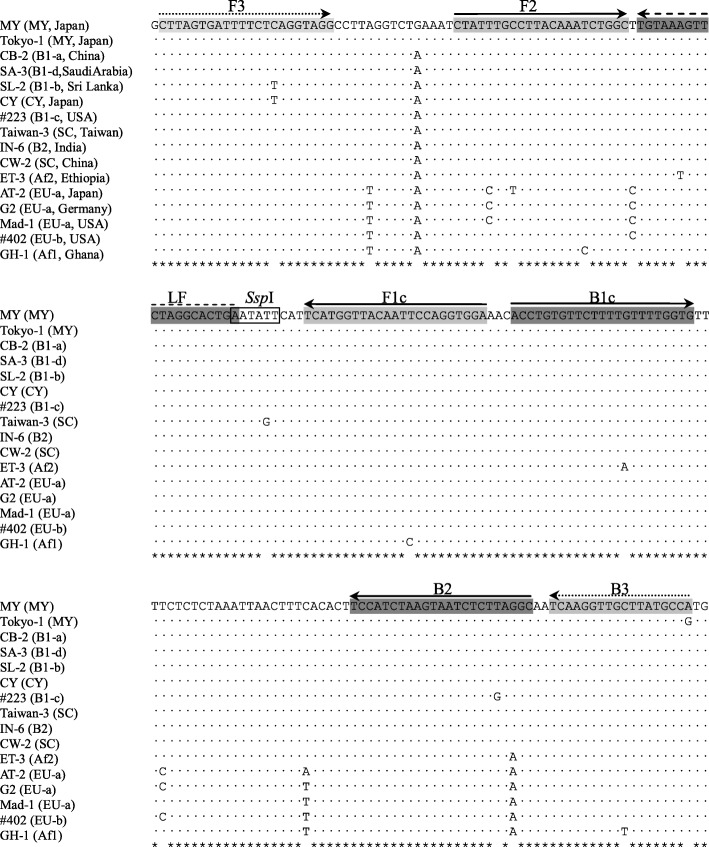
Alignment of partial region of the large T antigen gene. The nucleotide sequence of 16 JCV strains (nucleotide position 2984 to 3193, JCV MY strain) and the positions of the JCV LAMP primers are shown. The arrows indicate the locations of the primer target sequence and the directions of the primer extensions. The *Ssp*I restriction site is indicated by the box. JCV subtypes and country of origin of each strain based on the phylogenetic analysis [34] are indicated in parentheses. GenBank accession numbers for these strains in the alignment (top to bottom) are AB038250, AF030085, AB048550, AB048555, AB048553, AB038249, AF015532, U61771, AB048557, AB048579, AB048547, AB048569, AB038251, J02226, AF015528, and AB038252

Sixteen JCV strains were randomly selected from among 12 JCV subtypes [34], which are distributed worldwide throughout Europe, Africa, and Asia (Fig. 1). The partial sequences of the genome (VP1 to T antigen region) were aligned to identify the consensus sequence using the CLUSTAL-X 2.0 program, and the alignment files were applied to the LAMP primer design software Primer Explorer ver. 4 (Eiken Chemical Co., Tokyo, Japan). First, several sets of four primers were designed, comprising two outer primers (F3 and B3) and two inner primers (FIP and BIP). After the screening and selection of the four-primer set, an additional primer (LF) was designed using the same software.

### LAMP assay

The LAMP reaction was conducted with a Loopamp DNA amplification kit (Eiken Chemical Co., Ltd.) according to the manufacturer’s instructions. Briefly, the LAMP reaction mixture (25 μL) contained 40 pmol of FIP and BIP, 5 pmol of F3 and B3, and 20 pmol of LF, 2 × Reaction Mixture (12.5 μL), *Bst* DNA polymerase (1 μL), and the sample (2–5 μL). The reaction mixture was incubated at 63 °C for 60–120 min in a Loopamp real-time turbidimeter (LA-320C; Teramecs Co., Ltd., Kyoto, Japan), and then incubated at 80 °C for 5 min to terminate the reaction. Distilled water was used as a no template control (NTC). The LAMP assay results were assessed using the LA-320C software package (Teramecs Co., Ltd.). The cutoff turbidity value was fixed at 0.1 to differentiate positive from negative results.

Analysis of the LAMP products was conducted by 2% (*w*/*v*) agarose gel electrophoresis to verify specific band pattern and ascertained by restriction enzyme digestion with *Ssp*I (Nippon Gene, Toyama, Japan), which is a unique digestion site in the target sequence between F2 and B2 (Fig. 1). Visual fluorescence detection was performed by adding 10 μL of Gel Green™ dye (100 × solution in water; Biotium, Inc., Fremont, CA, USA) to each tube of the LAMP products and then visualized as fluorescent signals under a blue-light transilluminator Dark Reader™ (Clare Chemical Research, Inc., Delores, CO, USA) through an amber screen.

### Standard of quantitative LAMP assay

A 207 bp fragment was amplified by PCR from pJCV plasmid using the forward primer 5′-CTT AGT GAT TTT CTC AGG TAG GCC TTA GGT CTG AAA TCT ATT TGC CTT ACA AAT CTG G-3′ and the reverse primer 5′-TGG CAT AAG CAA CCT TGA TTG CCT AAG AGA TTA C-3′ to repair mismatch bases between the LAMP primer target sequence and the plasmid. The amplified fragment was cloned into the pGEM-T® Easy Vector system (Promega Corporation, Madison, WI, USA) using *E. coli* JM109 cells, and then the sequence of the insert was confirmed. The plasmid was digested with *Sca*I, and serially fivefold dilutions of purified DNA in EASY Dilution (for real-time PCR) (TakaRa Bio Inc., Shiga, Japan) were used for determining a detection limit and used as standards in the quantitative LAMP assays.

### Clinical CSF specimens

A total of 153 CSF specimens collected from 132 patients with suspected PML based on neurological symptoms and/or neuroimaging findings was used. These CSF specimens were sent to the Department of Virology 1, NIID, from the respective hospitals for routine testing for JCV genome by real-time quantitative PCR, as reported previously [13, 35]. Total DNA was extracted from 200 μL of the CSF specimens using the QIAamp DNA Blood Mini Kit (Qiagen) according to the manufacturer’s instructions. The extracted DNA was eluted to a final volume of 60 μL in buffer AE (Qiagen) and stored at − 30 °C until use.

Two μL of each sample DNA was tested twice in independent runs. Samples with at least one positive reaction within the 120 min reaction time of the LAMP assay were regarded as LAMP-positive. For quantification, the viral load was calculated using a standard curve drawn from the serially fivefold dilutions of the standard DNA (1.3 × 10^2^ to 2.0 × 10^6^ copies/reaction) in each run. When a value was calculated as less than 1 copy/reaction, that is, a logarithm of a negative number, the value was omitted. The cutoff value for quantification with the LAMP assay was set to 1.5 × 10^2^ copies/mL (equivalent of 1 copy/reaction). The average of the quantified values was considered as the viral load of the sample.

### Real-time quantitative PCR assay

The CSF specimens were tested for the detection and quantification of the JCV using the TaqMan-based quantitative real-time PCR (qPCR) assay as described previously [13]. Briefly, the qPCR primer set was targeted to the large T antigen gene. The qPCR assay was performed using 2 μL of each sample DNA prepared as described above and the cycling condition was 95 °C for 10 min, followed by 45 cycles of 95 °C for 10 s, 60 °C for 20 s, and 72 °C for 1 s.

### Statistical methods

The agreement between the LAMP and qPCR analyses was evaluated by kappa statistical analysis. Statistical difference in the JCV genome copy numbers in reaction between the LAMP negative and positives was tested by Mann-Whitney *U*-test. The correlation coefficient between the genome copy numbers in CSF determined by both assays was calculated using Pearson’s correlation coefficient.

### Ethical declaration

The study protocol was approved by the Ethical Committee for Biomedical Science of NIID (approval number 667). All the experiments were conducted in accordance with the ethical standards of the Declaration of Helsinki.

## Results

### Primer set screening and optimization of temperature for the JCV-LAMP assay

Seventeen sets of different primer combinations (4, 10, and 3 sets to target the VP1, large T antigen, and small t antigen genes, respectively) were screened for the ability to amplify the JCV genome sequence using pJCV (data not shown). The most efficient primer combination for JCV genome amplification was that targeted to the large T antigen gene (Fig. 1 and Table 1). The isothermal condition was optimized between 60 °C and 65 °C and the most effective reaction temperature was 63 °C. An additional loop-primer (forward loop primer LF) was subsequently designed and added to the primer set (Fig. 1 and Table 1).

**Table 1:** Sequences of the JCV-LAMP primers

Primer	Genome position^a^	Sequence (5′-3′)
F3	2985–3006	CTTAGTGATTTTCTCAGGTAGG
B3	3174–3191	TGGCATAAGCAACCTTGA
FIP (F1c + F2)	3073–3095, 3022–3043	TCCACCTGGAATTGTAACCATGA-CTATTTGCCTTACAAATCTGGC
BIP (B1c + B2)	3099–3121, 3149–3171	ACCTGTGTTCTTTTGTTTTGGTG-GCCTAAGAGATTACTTAGATGGA
LF	3045–3064	TCAGTGCCTAGAACTTTACA

^a^Genome position in the large T antigen gene of JCV MY strain (accession number: AB038250)

### Specificity of the JCV-LAMP assay

**Fig. 2:**
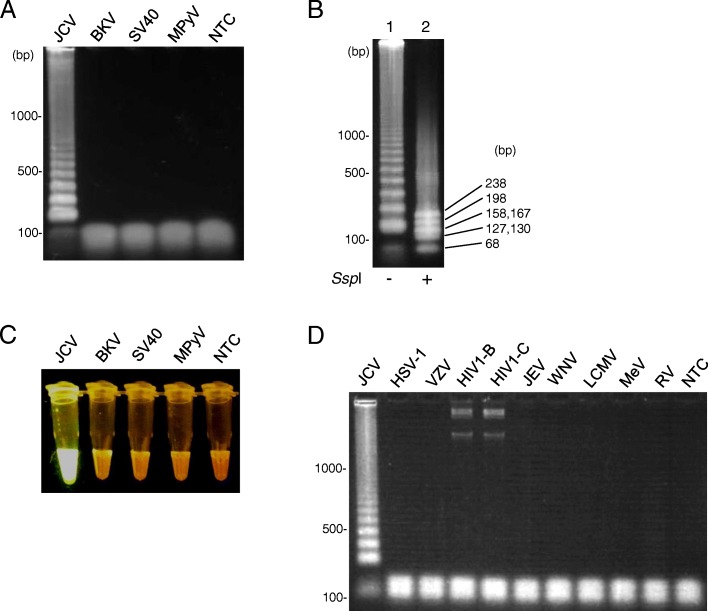
Cross-reactivity of JCV in the LAMP assay for JCV genome detection. **a** Agarose gel electrophoresis of the LAMP products performed with the polyomavirus DNAs (JCV, BKV, SV40, and MPyV) and no template control (NTC). **b** The LAMP-positive product (lane 1) and its *Ssp*I digested product (lane 2). **c** Visual detection of the LAMP products by adding nucleic acid staining reagent under the transilluminator. **d** The LAMP reaction was performed with viral DNA (HSV-1, VZV, and HIV type 1 subtype B and C) or RNA (JEV, WNV, LCMV, MeV, and RV) and an NTC. JCV, JC polyomavirus; BKV, BK virus; SV40, simian virus 40; MPyV, murine polyomavirus; HSV-1, herpes simplex virus type 1; VZV, varicella-zoster virus; HIV, human immunodeficiency virus; JEV, Japanese encephalitis virus; WNV, West Nile virus; LCMV, lymphocytic choriomeningitis virus; MeV, measles virus; RV, rabies virus

To evaluate the specific amplification of the JCV genome with the LAMP assay, the plasmid DNAs of polyomaviruses (including JCV, BKV, SV40, and MPyV) were tested as templates at a concentration of 1.0 × 10^7^ copies/reaction. After the LAMP reaction (63 °C for 120 min), the LAMP products were subjected to agarose gel electrophoresis. The JCV plasmid DNA showed a positive ladder pattern, as LAMP products consist of several inverted repeat structure, but the other polyomavirus plasmid DNAs including the NTC did not (Fig. 2a). Furthermore, the NTC was tested for the LAMP assays repeatedly 10 times and never showed a positive reaction. The mean turbidity value and standard deviations (SDs) calculated from the values of the 10 NTC was 0.012 ± 0.014, which was far below than the cutoff turbidity value of 0.1. In addition, by *Ssp*I digestion of the LAMP-positive product from the JCV plasmid DNA, the bands were converged to the sizes predicted of 68, 127, 130, 158, 167, 198, and 238 bp (Fig. 2b). Visual fluorescence detection of the LAMP products was also conducted by the addition of dye to each tube. Only the LAMP product from the JCV plasmid DNA was positive, as demonstrated by bright fluorescence (Fig. 2c), indicating that this detection method enables the differentiation of positive from negative reactions.

To evaluate cross-reactivity in the LAMP reaction against other viruses that may cause encephalitis, the viral DNA of HSV-1, VZV, HIV type 1 subtype B and subtype C, viral RNA of JEV, WNV, LCMV, MeV, and RV were subjected to the LAMP reaction at a concentration of not less than 1.0 × 10^7^ genome copies/reaction. As a result, only the sample from the PML patient (ID: P33), which included JCV genome confirmed by qPCR, showed a positive result (Fig. 2d). The other viral DNA (BKV, SV40, MPyV, HSV-1, VZV, and HIV) and RNA (JEV, WNV, LCMV, MeV, and RV) did not show any positive reactions in the JCV-LAMP method.

### Detection limit of the JCV-LAMP assay

**Fig. 3:**
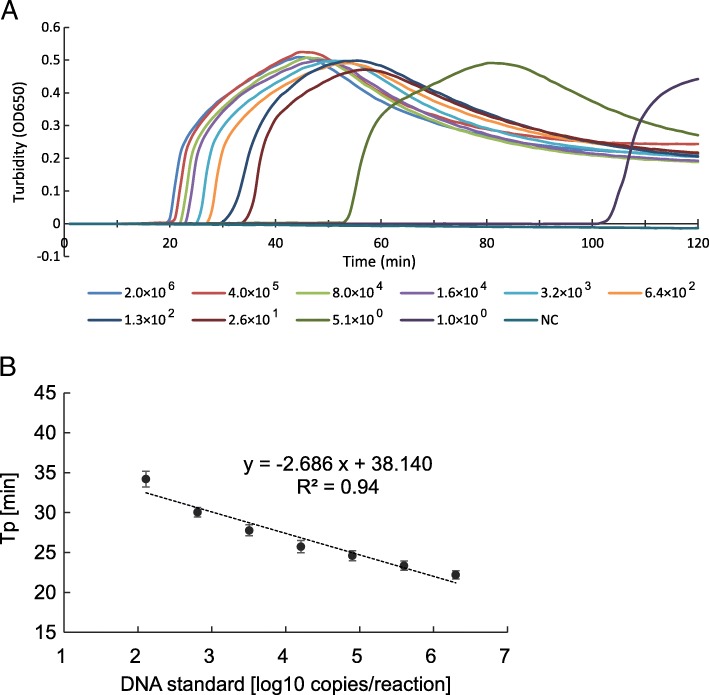
Amplification of the standard DNA in the LAMP assay. **a** Serially fivefold dilutions of standard DNA (from 1.0 × 10^0^ to 2.0 × 10^6^ copies/reaction) were subjected to the assay, and the LAMP products were monitored in real-time with a turbidimeter. Standard diluent was used as a negative control (NC). **b** A standard curve for the quantitative LAMP assay. The time to a positive result (Tp) was plotted against input standard DNA (from 1.3 × 10^2^ to 2.0 × 10^6^ copies/reaction). The results are the average of four replicates. Error bars represent the standard deviation (SD)

To examine the detection limit of the LAMP assay, standard DNA diluted fivefold serially from 1.0 × 10^0^ to 2.0 × 10^6^ copies/reaction were used as a template, and the time to a positive result (Tp), at which the turbidity value was greater than 0.1, was measured by real-time turbidimeter. The LAMP assay could detect 1.0 × 10^0^ copy of the standard DNA within 120 min (Fig. 3a). A standard curve between Tp and the copy number of standard DNA ranging from 1.3 × 10^2^ to 2.0 × 10^6^ copies/reaction was generated in quadruplicate (Fig. 3b). The standard curve showed a high degree of linearity between the Tp and the copy number of standard DNA (*R*^2^ = 0.94).

### Efficacy of the JCV-LAMP assay in JCV genome detection with using clinical specimens

The LAMP assay was performed twice using 2 μL of extracted DNA from CSF specimens, because of the sample amount limitation. Of the 153 specimens from patients with suspected PML, the reactions of 45 (29%) were positive in the LAMP assay, which included 42 of the 50 qPCR-positive specimens and 3 of the 103 qPCR-negative specimens (Table 2). The amplified LAMP products from three qPCR-negative specimens were found to have originated from the JCV genome and not the false-positive results by the agarose gel electrophoresis analysis after *Ssp*I restriction enzyme treatment and the amplification curve monitored by real-time turbidimeter (data not shown). The sensitivity, specificity, positive predictive value, and negative predictive value of the LAMP assay were 84% (42/50), 97% (100/103), 93% (42/45), and 93% (100/108), respectively, as calculated with the qPCR assay-based data as the standard. Statistical analysis showed a nearly perfect agreement (Kappa = 0.83) between the LAMP and qPCR analyses of the clinical specimens (Table 2).

**Table 2:** Results of JCV detection in clinical specimens with the LAMP assay and qPCR

LAMP assay	qPCR assay	
	Positive	Negative
Positive	42	3
Negative	8	100

**Fig. 4:**
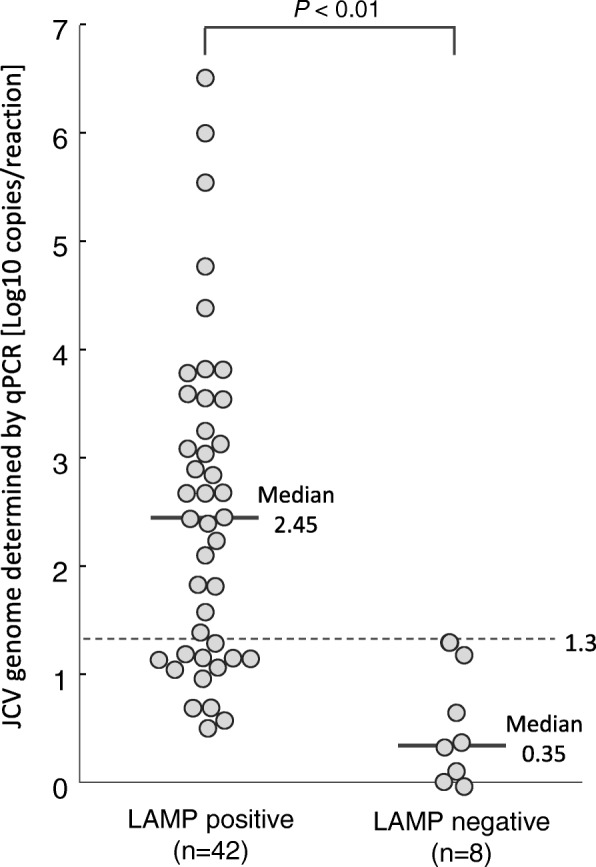
Relationship between the LAMP results and the JCV genome copy numbers of samples. The assay was performed twice independently using 2 μL of extracted DNA with the qPCR-positive (*n* = 50). Circles show the JCV genome copy number of the sample per reaction determined by the qPCR. The vertical lines and values indicate the median values of the JCV genome copy number of the samples per reaction for each result group. The dashed line indicates the 100% detection limit of log_10_1.3 (=20) copies/reaction

The relationship between the LAMP results and the level of JCV copy numbers of samples, as measured by qPCR, are shown in Fig. 4. Among the 50 qPCR-positive specimens, 42 containing 3.2 × 10^0^ to 3.2 × 10^6^ copies of the JCV genome/reaction showed positive reactions. Eight specimens containing 0.9 to 19.9 copies/reaction were LAMP-negative. The levels of JCV DNA in the LAMP-negative group (median: 2.2 copies/reaction) were significantly lower than those found in LAMP-positive group (median: 2.8 × 10^2^ copies/reaction) (*p* < 0.05). As seen in Fig. 4, the sensitivity of the specimens containing equal to or more than 20 × 10^1^ copies in 2 μL of extracted DNA (equivalent to ≥3.0 × 10^3^ copies/mL CSF) was 100% (29/29), whereas that of the specimens containing less than 20 copies/reaction was 62% (13/21).

**Fig. 5:**
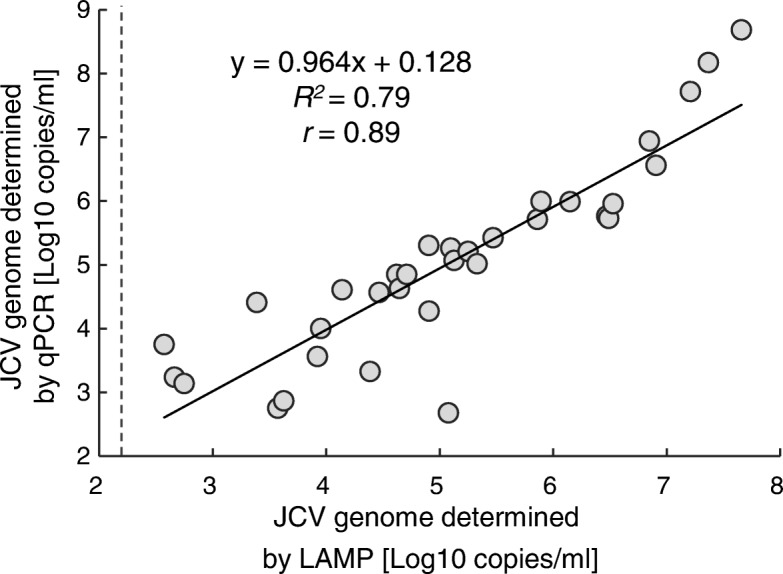
Correlation between the LAMP and qPCR quantitative analyses. The JCV genome copy numbers, as determined by the LAMP assay (x-axis) vs. qPCR (y-axis). Among the 42 LAMP- and qPCR-positive samples, 33 samples, for which the viral genome was quantified as ≥1 copies/reaction (corresponds to ≥1.5 × 10^2^ copies/mL of CSF) by the LAMP assay, were subjected to the analysis (*n* = 33). The dashed line indicates the cutoff value for the quantification with the LAMP assay of 1.5 × 10^2^ copies/mL of CSF

Among 42 LAMP- and qPCR-positive specimens, 33 were quantified by the LAMP assay and 9 specimens, for which the viral load was lower than the cutoff value for the quantification, were excluded from analysis. A positive correlation was demonstrated between the copy numbers determined by the LAMP assay and those determined by the qPCR assay for 33 LAMP- and qPCR-positive specimens (*r* = 0.89, *p* < 0.05) (Fig. 5). For the nine unquantified specimens, positive results were obtained after 45 min of reaction time and the calculated viral copy number was less than 1 copy/reaction. However, those 9 specimens should be supposed to contain JCV genome (median 1.4 × 10^1^, interquartile range: 1.4 × 10^1^ to 2.0 × 10^1^ copies/reaction) in the quantification of qPCR.

**Fig. 6:**
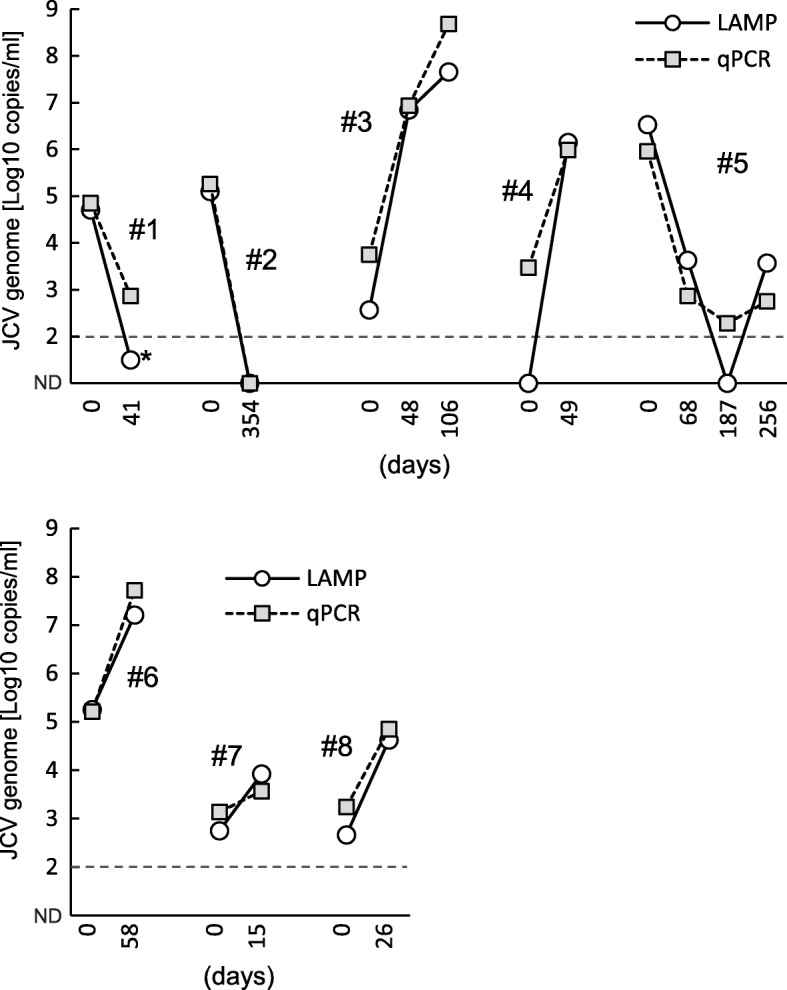
Changes in the JCV genome copy number in CSF in sequential samples. Underlying diseases and condition of eight PML patients (#1 to #8) are as follows: two patients had HIV/AIDS (#1 and #2) and received antiretroviral therapy (HAART) after the first CSF sampling and four had hematologic disorders (acute lymphoblastic leukemia with bone marrow transplantation (#3), chronic lymphocytic leukemia (#4), acute myeloid leukemia with umbilical cord blood transplantation (#5), and primary macroglobulinemia (#6)), one had an autoimmune disorder (systemic lupus erythematosus) (#7), and one had hepatitis C virus-related liver disease (#8). Circles show the JCV genome copy numbers, as determined by the LAMP assay, and squares show those determined by the qPCR assay. Asterisk indicates the sample which showed LAMP-positive but the viral genome copy number was lower than cut off value in the LAMP quantification. Days counted from the first CSF sampling date. ND, not detected

Transition of the JCV genome load in the CSF samples of eight PML patients is shown in Fig. 6. For two HIV/AIDS (acquired immunodeficiency syndrome) patients (#1 and #2), the JCV load decreased at the second sampling, when HAART had already been initiated. An increase and decrease was seen in case #5, as previously reported [36]. On the other hand, the other patients showed an increase in JCV load within a few months (#3, #4, #6, #7, and #8).

## Discussion

A JCV-LAMP assay developed in the present study is considered to be an efficacious tool for diagnosis of PML, although it takes a rather longer time than qPCR. The diagnosis of PML should not rely only on the virological tests. The JCV-LAMP assay developed had high sensitivity and specificity based on the highly sensitive qPCR. Three of the 103 qPCR-negative specimens showed a positive reaction in the LAMP assay (Table 2). These three CSF specimens contained JCV genome, and the positive result was confirmed to be evident with the agarose gel electrophoresis analyses as well as the consideration of their clinical backgrounds. Two of these patients were HIV positive, and the other had multiple sclerosis treated with steroid therapy. HIV associated PML cases accounts for approximately 20% of all cases in Japan [35].

The measurement of the JCV load in CSF is valuable not only for diagnosis but also for clinical management and prediction of the prognosis for PML patients [17]. For instance, the patient #5 (Fig. 6) developed PML following a umbilical cord blood transplant [36]. This patient showed a favorable response to an experimental therapy with mefloquine, which inhibits JCV replication in vitro [37], although the efficacy of mefloquine in the treatment of PML is not conclusive. In addition, the JCV load in PML patients fluctuates over a rather brief period (Fig. 6); thus, repeated sample collection and testing is required, especially from patients suspected of having PML based on the patients’ clinical backgrounds.

Since PML was reported as an adverse drug event in patients administered with the therapeutic immune-modulatory monoclonal antibodies, such as rituximab, natalizumab, and efalizumab [38, 39], it is suggested that the incidence of PML may increase in the near future. The patients treated with these immune-modulatory monoclonal antibodies should also be monitored closely for PML. The JCV-LAMP assay may become a useful and efficacious tool for monitoring PML for such patients.

The detection limit of the JCV-LAMP assay was higher than that of the qPCR. The detection limit should be lowered to increase the sensitivity of the assay. In this study, 2 μL of extracted DNA from CSF were used as a template. It would be likely that the detection limit can be lowered by using 5 μL-template in a 25 μL reaction mixture. Furthermore, a LAMP reaction is hardly affected by PCR inhibitors. In this study, DNAs extracted from CSF using the extraction kit were used as a template. The usefulness of the JCV-LAMP assay using CSF sample without DNA extraction processing should be evaluated. The target genome in urine sample can be successfully amplified in a LAMP reaction without DNA extraction processing [31]. So, further studies for making the JCV-LAMP assay more usefulness in clinical settings and more sensitive are required.

The LAMP reaction requires no denaturing step or thermal cycling in the reaction process; therefore, LAMP can be performed using relatively simple equipment, such as a heating block or water bath and also be employed by visual examination. Thus, the LAMP assay can be a useful diagnostic tool for the diagnosis of PML in countries with resource-limited setting where MRI or PCR machines are not installed adequately. Although the LAMP primer was designed to detect various kinds of JCV subtypes (Fig. 1), only clinical specimens obtained in Japan were evaluated in this study, where CY and MY are major JCV subtypes [40]. Therefore, the reaction against JCV circulating in the other parts of the world should be tested.

## Conclusion

A quantitative JCV-LAMP assay useful not only for the diagnosis of but also for the prognostic prediction of patient with PML was developed.

## Abbreviations

BKV: BK virus
CSF: Cerebrospinal fluid
HAART: Highly active antiretroviral therapy
HIV: Human immunodeficiency virus
HSV-1: Herpes simplex virus type 1
IQR: Interquartile range
JCV: JC polyomavirus
JEV: Japanese encephalitis virus
LAMP: Loop-mediated isothermal amplification
LCMV: Lymphocytic choriomeningitis virus
LP: Loop-primer
MeV: Measles virus
MPyV: Murine polyomavirus
MRI: Magnetic resonance imaging
NTC: No template control
PML: Progressive multifocal leukoencephalopathy
qPCR: Real-time quantitative PCR
RV: Rabies virus
SD: Standard deviation
SV40: Simian virus 40
Tp: Time to a positive result
VZV: Varicella-zoster virus
WNV: West Nile virus
