# Correction to: A loop-mediated isothermal amplification assay for the detection and quantification of JC polyomavirus in cerebrospinal fluid: a diagnostic and clinical management tool and technique for progressive multifocal leukoencephalopathy

**DOI:** 10.1186/s12985-018-1057-9

**Published:** 2018-10-08

**Authors:** Hitomi Kinoshita, Kazuo Nakamichi, Chang-Kweng Lim, Mutsuyo Takayama-Ito, Lixin Wang, Itoe Iizuka, Ichiro Kurane, Masayuki Saijo

**Affiliations:** 1 Department of Virology 1, National Institute of Infectious Diseases, Shinjuku-ku, Tokyo, 162-8640 Japan; 2 Present Address: School of Tropical and Laboratory, Hainan Medical University, Haikou, 571199 Hainan China

## Correction

In the original publication of article [1], ‘20 × 10^1^ copies’, which is in the sentence ‘*As seen in Fig. 4, the sensitivity of the specimens containing equal to or more than 20 × 10*^*1*^ *copies in 2 μL of extracted DNA (equivalent to ≥3.0 × 103 copies/mL CSF) was 100% (29/29)*’, changes to ‘2.0 × 10^1^ copies’ in results section. The publisher apologizes to the readers and authors for the inconvenience.

The original article has been corrected.
